# Anaphylaxis in Schools: Results of the EPIPEN4SCHOOLS Survey Combined Analysis

**DOI:** 10.1089/ped.2016.0675

**Published:** 2016-09-01

**Authors:** Martha V. White, Susan L. Hogue, Dawn Odom, Darryl Cooney, Jennifer Bartsch, Diana Goss, Kelly Hollis, Christopher Herrem, Suyapa Silvia

**Affiliations:** ^1^ Institute for Asthma and Allergy, Wheaton, Maryland.; ^2^ RTI International, Research Triangle Park, North Carolina.; ^3^ Mylan Specialty L.P., Canonsburg, Pennsylvania.

## Abstract

A pilot survey described the characteristics of anaphylactic events occurring in an initial set of participating U.S. schools during the 2013–2014 school year. This survey was subsequently readministered to large school districts, which were underrepresented in initial results. A cross-sectional survey was administered to the U.S. schools that were participating in the EPIPEN4SCHOOLS^®^ program (Mylan Specialty L.P., Canonsburg, PA) to assess characteristics of anaphylactic events. Data from large school districts were added to initial findings in this comprehensive combined analysis. A total of 1,140 anaphylactic events were reported among 6,574 responding schools. Of 1,063 anaphylactic events with data on who experienced the event, it was observed that it occurred mostly in students (89.5%, 951/1,063). For students, anaphylactic events were reported across all grades, with 44.9% (400/891) occurring in high school students, 18.9% (168/891) in middle school students, and 32.5% (290/891) in elementary school students. Food was identified as the most common trigger (60.1%, 622/1,035). A majority of schools (55.0%, 3,332/6,053) permitted only the school nurse and select staff to administer epinephrine to treat anaphylaxis. The unpredictability of anaphylaxis is emphasized by its high occurrence in individuals with no known allergies (25.0%). A majority of schools permitted only the school nurse and select staff to treat anaphylaxis. Thus, individuals experiencing an anaphylactic event may frequently encounter staff members not being permitted to administer potentially life-saving epinephrine. Epinephrine auto-injectors provided by the EPIPEN4SCHOOLS program were used to treat 38.0% of events. Anaphylaxis can occur in children with no previously known allergies, illustrating the importance of public access to epinephrine.

## Introduction

Anaphylaxis is a serious acute reaction that is potentially fatal.^1^ Guidelines recommend intramuscular injections of epinephrine as first-line therapy for anaphylaxis.^2,3^ Delayed administration of epinephrine is associated with fatalities,^4^ and early use of epinephrine is associated with a decreased risk of hospitalization.^5^ In the community setting, epinephrine can be promptly self-administered, or administered by a caregiver, by using an epinephrine auto-injector (EAI).^1^ As the prevalence of food allergies increases,^6^ ensuring the availability of EAIs in the community becomes increasingly important. A growing area of concern is the preparedness of schools to both recognize and treat anaphylaxis.^7^

In 2012, Mylan Specialty L.P. launched the EPIPEN4SCHOOLS^®^ program (Canonsburg, PA) to provide EpiPen^®^ (epinephrine injection) Auto-Injectors and educational materials to qualifying schools. More than 50,000 public and private schools across the United States have participated in this program. In an effort to collect data on anaphylactic events that occur in schools, an initial survey of 6,019 schools participating in the EPIPEN4SCHOOLS program was conducted; the data collection period for this initial survey was from May 21, 2014, to July 9, 2014.^8,9^ Results from this survey showed that more than 1 in 10 responding schools reported an anaphylactic event during the 2013–2014 school year.^8,9^ Anaphylactic events occurred in students across all grade levels, with almost 50% occurring in high school students.^9^ Notably, 22% of these events were experienced by individuals with no known allergies, and ∼25% of those who suffered an anaphylactic attack were not treated with epinephrine. These initial results demonstrated the need for schools to stock EAIs and to provide resources for training school personnel on the proper treatment of anaphylaxis.

It is important to note, however, that schools in the largest districts were underrepresented in these initial results. Many of the larger school districts collect data on anaphylactic events, but the data are typically available only in aggregate district-level format. In addition, identifying the correct contact person to provide data and obtaining approval to collect data through the various research application processes was a challenge. Thus, the large school district information was underrepresented in the data obtained during the initial data collection period. In this article, school-level data from larger school districts have been added to data obtained during the initial data collection period to develop a comprehensive, combined, school-level analysis.

## Materials and Methods

### Data source

A cross-sectional pilot survey of U.S. schools that participated in the EPIPEN4SCHOOLS program during the 2013–2014 academic year was conducted. The survey consisted of 16 questions, as previously described.^9^ The person best suited to respond regarding all occurrences of severe allergic reactions and treatments administered at each school completed the survey. The effective sample for the survey consisted of 32,387 public and private schools for which there was school-level contact information available. A database of schools that have participated in the EPIPEN4SCHOOLS program is maintained by BioRidge Pharma, which provided logistical mailing services during administration of the survey. Methods for collecting data from 6,019 schools that responded to an initial web-based survey between May 21, 2014, and July 9, 2014, have been previously described in greater detail.^8^

A second data collection period was completed between October 2014 and January 2015 to obtain data from the 60 largest participating school districts. For this data collection period, each district was directly contacted by telephone and e-mail. An initial point of discussion was the timing of and requirements for completing research applications to obtain approval for conducting research in those districts. A questionnaire identical to the one used for the initial data collection survey was distributed, and data received via e-mail from district contacts or surveys sent via e-mail or fax directly from schools were entered into a database.

The study was submitted to the RTI institutional review board (IRB) for approval, which determined that the research activity did not constitute research involving human subjects as defined by the US Code of Federal Regulations (45 CFR 46.102). The approval of these activities by the RTI IRB was not necessary; therefore, an exemption was granted.

### Data analysis

The methods for the combined analysis paralleled those of the 2 separate analyses for each data collection method and incorporated data from both. Data collected during the initial collection period were submitted by individual schools and analyzed as previously described.^8^ In the second, large-district data collection, data were submitted in a variety of formats. Some districts responded with a single aggregate response that represented all schools within that district. In other districts, a subset of individual schools completed the surveys, providing school-level information. Some districts provided a combination of individual and aggregate responses. Only those responses from districts that provided school-level information were used in this analysis. Aggregate data collected at the district level were not included.

The combined analysis pooled school-level data from the initial collection period with data collected during the second collection period. For schools that were located in large districts that responded to the first survey but did not respond to the second one, the data from the first collection were used in the combined analysis. In addition, for schools that responded to both surveys, the most recent data were used in the combined analysis.

Characteristics of participating schools (eg, region, grade level, type and source of stocked EAIs) and of anaphylactic events (individual who experienced the event, known allergies, trigger that caused the event, treatment given) were reported by using descriptive statistics. Additional descriptive analyses of characteristics of participating schools included school staff who were trained to recognize anaphylaxis and those who were allowed to administer epinephrine to treat anaphylaxis. Relative frequency was calculated by dividing the total number of responses for each response category for a variable of interest by the combined number of total responses (including those that the respondent marked as “unknown”). Missing responses were excluded from the analysis.

## Results

### Survey completion

A total of 6,019 surveys were submitted during the initial data collection period,^8^ and 608 school-level responses were obtained from schools in large districts during the second data collection period. The total number of responses used in this combined analysis, after removal of 53 duplicate responses, was 6,574. When comparing the results of the 53 schools that responded to both surveys, 67.9% (*n* = 36) reported the same number of anaphylactic events in both surveys. The position of the respondent was reported for 6,447 of the surveys submitted. Of these, 77.5% (*n* = 4,999) were completed by the school nurse, 8.4% (*n* = 543) by other health staff, and 14.0% (*n* = 905) by other school staff.

### Characteristics of anaphylactic events

**FIG. 1.**
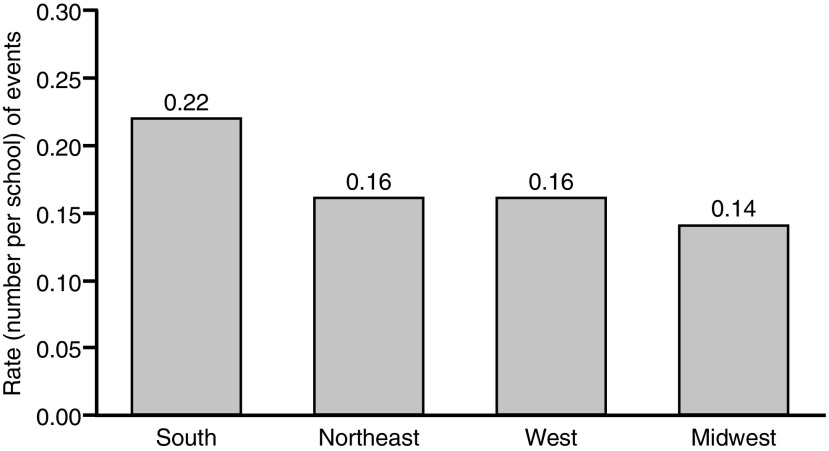
Rate of anaphylactic events among responding schools by region. The rate is calculated as the number of events in each region divided by the number of schools for that specific region. Multiple events could be reported for a single school. The event rate per school does not account for the total number of students within each school.

**FIG. 2.**
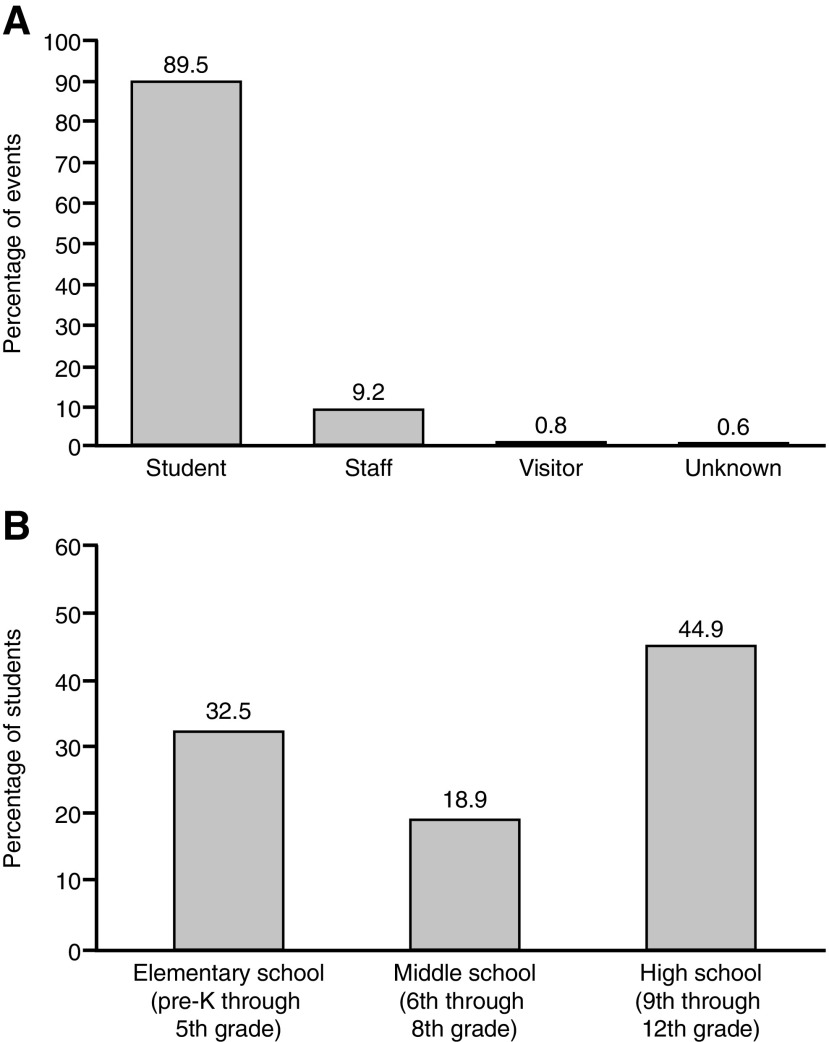
Distribution of anaphylactic events by **(A)** who had the attack and **(B)** school grade of students. pre-K, pre-kindergarten.

A total of 1,140 anaphylactic events were reported in 736 schools, with some schools reporting multiple events. Of the responding schools, those in the South reported the highest rate of events, with 0.22 events per school (*n* = 354), followed by those in the Northeast with 0.16 events per school (*n* = 477), and those in the West with 0.16 events per school (*n* = 88; Fig. 1). Schools in the Midwest reported 0.14 events per school (*n* = 221). Data regarding the person who had the attack were available for 1,063 events. Of these, 89.5% (*n* = 951) occurred in students, 9.2% (*n* = 98) occurred in staff, 0.8% (*n* = 8) occurred in visitors, and 0.6% (*n* = 6) occurred in individuals whose status was unknown (Fig. 2A). The school grade of the student who had an anaphylactic event was available for 891 events. Of these, 32.5% (*n* = 290) occurred in elementary school students (pre-kindergarten [pre-K] through 5th grade), 18.9% (*n* = 168) occurred in middle school students (6th through 8th grade), and 44.9% (*n* = 400) occurred in high school students (9th through 12th grade; Fig. 2B).

### Allergy history and anaphylactic triggers

Of the 1,049 reported anaphylactic events with information available on allergy history, 68.1% (*n* = 714) occurred in individuals with a known allergy, whereas 25.0% (*n* = 262) occurred in individuals without a known allergy. Individual allergy history for 7.0% of events (*n* = 73) was unknown. The number of events that occurred in individuals with a known allergy was similar across grade levels, with no apparent trend (Table 1). Of the events that occurred in elementary school students, 24.6% (*n* = 71) were in individuals with no known allergy, which was comparable to the rates observed for middle school students (*n* = 47; 28.0%) and high school students (*n* = 74; 18.6%).

**Table 1.** Known Allergies for Students Who Had an Anaphylactic Event by Grade Level

	*Number of events,* n/N^a^*(%)*		
*Grade level*	*Known allergy*	*No known allergy*	*Allergy status unknown*
Elementary school	212/289 (73.4)	71/289 (24.6)	6/289 (2.1)
Middle school	113/168 (67.3)	47/168 (28.0)	8/168 (4.8)
High school	288/398 (72.4)	74/398 (18.6)	36/398 (9.0)

^a^ *N* excludes missing data.

**FIG. 3.**
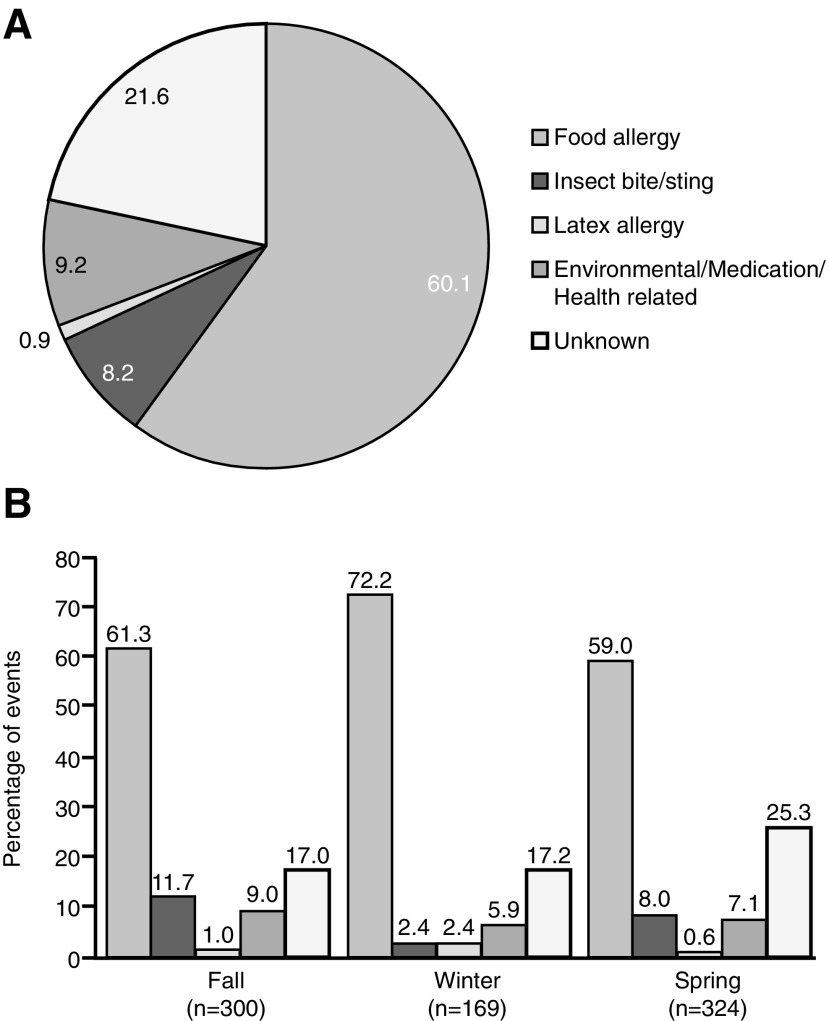
Percentage of triggers for anaphylactic events **(A)** overall and **(B)** in students distributed by season.

Triggers were identified in 78.4% (*n* = 811) and unknown in 21.6% (*n* = 224) of the 1,035 events for which data on triggers were available. Food was the most common trigger, having been identified in 60.1% of events (*n* = 622). In addition, 8.2% of triggers (*n* = 85) were reported as insect bites or stings; 9.2% (*n* = 95) as environmental, medication, or health related; and 0.9% (*n* = 9) as latex (Fig. 3A).

Data were available on the seasonality of anaphylactic triggers for the 951 events that occurred in students (Fig. 3B). Overall, fewer events occurred in the winter (*n* = 169) than in the fall (*n* = 300) or spring (*n* = 324). Food was the predominant trigger across all seasons, whereas other triggers varied by season. Of note, insect bites/stings were identified as the trigger for 11.7% of events that occurred during the fall (*n* = 35), for 8.0% of those that occurred during the spring (*n* = 26), and for only 2.4% of those that occurred during the winter (*n* = 4).

### Treatment of anaphylaxis

**FIG. 4.**
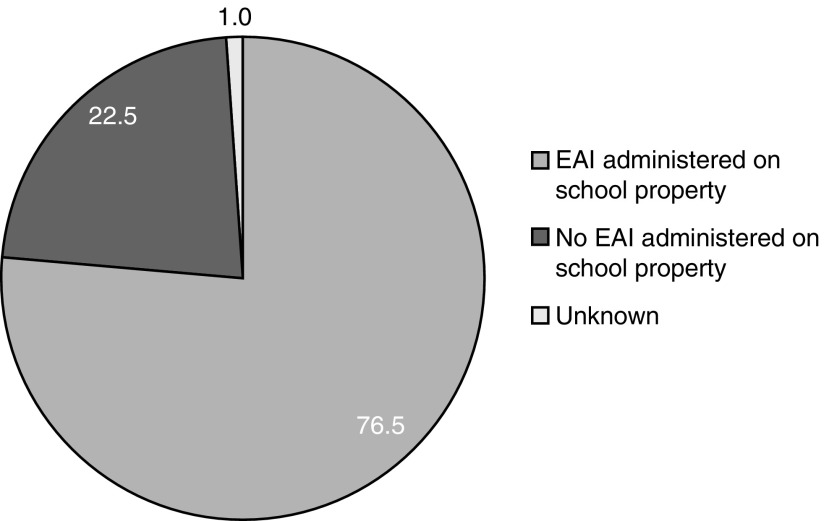
Percentage of EAIs administered on school property. EAI, epinephrine auto-injector.

Data on use of EAIs on school property were available for 1,059 events (Fig. 4). EAIs were administered in 76.5% of these anaphylactic events (*n* = 810), whereas EAIs were not administered in 22.5% of events (*n* = 238). It was unknown whether EAIs were administered in the remaining 1.0% of events (*n* = 11).

Of the 1,012 anaphylactic events with data on type of treatment given, 38.0% (*n* = 385) were treated with stock EpiPen Auto-Injectors from the EPIPEN4SCHOOLS program, 33.7% (*n* = 341) were treated with a personal EpiPen Auto-Injector, 2.9% (*n* = 29) were treated with another type of EAI, 17.6% (*n* = 178) were treated with an antihistamine, and 3.4% (*n* = 34) were treated with another alternative therapy (Table 2). No therapy was administered in 2.0% of these events (*n* = 20).

**Table 2.** Source and Type of Treatment of Anaphylactic Events (*N* = 1,012)

*Type of treatment*	*Number of events,* n *(%)*
School stock EpiPen^®^ Auto-Injector	385 (38.0)
Personal EpiPen Auto-Injector	341 (33.7)
Other type of EAI	29 (2.9)
Unknown EAI	9 (0.9)
Antihistamine	178 (17.6)
Other treatment	34 (3.4)
Unknown treatment	5 (0.5)
No treatment given	20 (2.0)
Unknown whether EAI was administered	11 (1.1)

EAI, epinephrine auto-injector.

### Training and administration of epinephrine by school staff

**FIG. 5.**
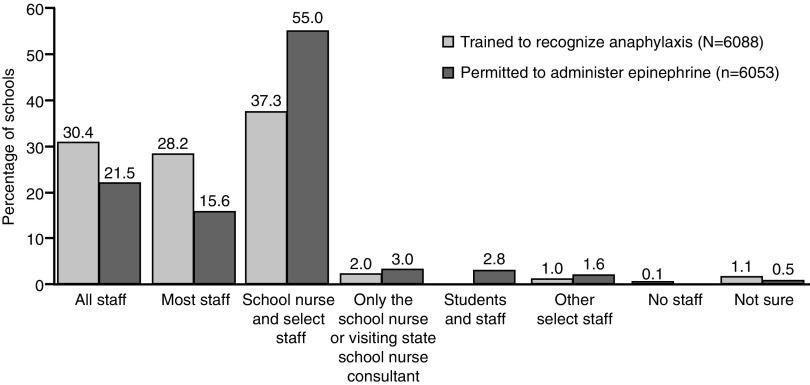
School staff trained to recognize the symptoms of anaphylaxis and permitted to administer epinephrine.

A total of 6,088 responding schools provided data on staff training for anaphylaxis recognition. Of these, 30.4% (*n* = 1,851) provided training for all staff, 28.2% (*n* = 1,717) for most staff, 37.3% (*n* = 2,268) for the school nurse and select staff, and 2.0% (*n* = 120) for only the school nurse (Fig. 5).

In addition, 6,053 responding schools provided data regarding who is permitted to administer epinephrine to treat anaphylaxis. Of these, 21.5% (*n* = 1,300) permitted all staff, 15.6% (*n* = 942) permitted most staff, 55% (*n* = 3,332) permitted the school nurse and select trained staff, and 3.0% (*n* = 181) permitted only the school nurse to administer epinephrine to treat anaphylaxis (Fig. 5).

## Discussion

The data presented in this combined analysis support the idea that anaphylaxis is not uncommon in U.S. schools,^10^ and they are consistent with results obtained during the initial data collection period.^8^ More than 10% of responding schools reported at least 1 anaphylactic event during the 2013–2014 school year, most of which were experienced by students (89.5%). Although anaphylactic events were reported across all grades, they occurred most commonly in high school students. This is consistent with reports showing that individuals aged between 15 and 19 years are more likely to be hospitalized for anaphylactic attacks than those aged between 5 and 14 years.^11^ Adolescents and young adults with known food allergies are regularly in unsupervised social situations where they may feel pressured to engage in risky behaviors, with more than 50% admitting to eating known allergens.^12^ Since food was the predominant trigger in the combined EPIPEN4SCHOOLS survey, this may partially explain why high school students experienced more anaphylactic events. In addition, 25% of events occurred in individuals with no history of allergies, a trend seen across all grade levels, demonstrating the importance of broad public access to epinephrine. Thus, it is important to provide adequate education to all children and those supervising them (eg, teachers, other school staff) about the triggers, consequences, and treatment of severe allergic reactions.

Generally accepted guidelines recommend prompt administration of epinephrine for the treatment of anaphylaxis.^2,3^ In accordance with these guidelines, EAIs were administered at responding schools in more than 75% of the reported anaphylactic events. Notably, 38.0% of all anaphylactic events were treated with an EpiPen Auto-Injector provided by the EPIPEN4SCHOOLS program. However, 17.6% of anaphylactic events were treated with an antihistamine in lieu of an EAI, despite the fact that antihistamines do not treat the most serious symptoms of anaphylaxis and are to be used only as adjunctive therapy.^13^ This suggests that although programs designed to distribute and stock EAIs in the community setting, such as the EPIPEN4SCHOOLS program, seem to be beneficial and important, there may still be room for improvement in the training of school staff, as well as opportunities to improve school policies regarding the recommended first-line treatment of anaphylaxis.

Survey findings suggest that there may be an opportunity to improve school staff training programs. Only 58.6% of schools surveyed trained all or most staff members to recognize the signs and symptoms of anaphylaxis. Similarly, only 37.0% of responding schools permitted all or most staff to administer epinephrine. These data suggest that students may often encounter staff members who are not trained to recognize anaphylaxis or administer epinephrine, potentially delaying treatment. Since early use of epinephrine is associated with improved clinical outcomes^5^ and delayed administration of epinephrine is associated with fatalities,^4^ school policies should be designed to allow for prompt administration of epinephrine during the early stages of an anaphylactic attack.

Though consistent with results obtained during the initial data collection period,^8^ the results of this combined analysis, which includes schools in large districts, provide a more comprehensive representation of anaphylaxis in U.S. schools. These data may be used to help create awareness of the importance of training for the recognition of anaphylaxis and epinephrine administration in schools and to support the need for public access to epinephrine.

### Study limitations

In general, surveys are subject to a number of measurement errors, including systematic and random variance from the respondents, such as failure to carefully read a question or misreporting an event. Specifically, in this survey, one of the response options for trigger type was “unknown,” which could mean that either the respondent did not have the information or the individual experiencing the event was actually unaware of what triggered the event. The data are also limited by variations in the level of detail on anaphylactic events that is recorded at the schools and may be subject to a respondent's recollection of the events.

Surveys have the potential for response bias if the respondents do not accurately represent a cross-section of the target population. It is possible that schools with anaphylactic events may have been more likely to respond to the survey, whereas schools with no anaphylactic events may have been less likely to respond, thus potentially overestimating the rates of anaphylaxis.

Some districts that responded in the second, large-district data collection period could only provide aggregate district-level data. To provide a level of analysis comparable to that used in the initial data collection period, only school-level responses were used in this article. This led to the exclusion of 35 district-level responses from large school districts.
